# Short-term efficacy of ticagrelor in acute ST-segment elevation myocardial infarction patients undergoing an emergency percutaneous coronary intervention

**DOI:** 10.18632/aging.102353

**Published:** 2019-10-30

**Authors:** Bangming Cao, Fuzheng Qu, Xianliang Liu, Changzheng Gao, Qiang Fu, Chunying Jiang, Peng Wei, Qianqian Ma

**Affiliations:** 1 Department of Cardiology, Yantai Affiliated Hospital of Binzhou Medical University, Yantai 264100, Shandong, China; 2 Department of Cardiology, Affiliated Hospital of Jiangnan University, Wuxi 214062, China; 3 Department of Cardiology, Xuzhou Central Hospital, The Affiliated Hospital of Southeast University, Xuzhou 221009, Jiangsu, China; 4 XuZhou Clinical School of Xuzhou Medical University, Xuzhou 221009, Jiangsu, China; 5 Department of Rheumatology, Xuzhou Central Hospital, The Affiliated Hospital of Southeast University, Xuzhou 221009, Jiangsu, China

**Keywords:** ticagrelor, clopidogrel, ST-segment elevation myocardial infarction, percutaneous coronary intervention, TIMI blood flow

## Abstract

In total, 97 acute ST-segment elevation myocardial infarction (STEMI) patients who received an emergency percutaneous coronary intervention (PCI) were enrolled and divided into a ticagrelor group and a clopidogrel group. Thrombolysis in myocardial infarction (TIMI) blood flow and the corrected TIMI frame count (CTFC) were used to assess the blood perfusion of culprit vessels. Thromboelastography (TEG) was used to evaluate the antiplatelet effect of drugs. The results showed that the incidence of TIMI grade III blood flow in the ticagrelor group was significantly higher than that in the clopidogrel group. The CTFC in the anterior descending, circumflex, and right coronary arteries was statistically significantly lower in the ticagrelor group as compared with that in the clopidogrel group. At 2 h and 7 d postdrug treatment, the adenosine diphosphate-induced platelet inhibition rate (ADP%) in the ticagrelor group increased significantly as compared with that in the clopidogrel group, and the platelet aggregation rate of the ADP pathway (MA_ADP_) decreased significantly in the ticagrelor group versus that in the clopidogrel group. In conclusion, ticagrelor significantly improved TIMI blood flow and had a better antiplatelet effect than clopidogrel in STEMI patients undergoing an emergency PCI.

## INTRODUCTION

With improvements in people’s living standards and changes in diets and lifestyles, there have been marked changes in the disease spectrum, with cardiovascular diseases now a leading cause of death and disability. About 20 million people worldwide die each year of cardiovascular diseases [1–3]. In China, the toll of cardiovascular-related illnesses is particularly severe, with an estimated 290 million cardiovascular patients, accounting for 44.8% and 41.9% of total deaths in rural and urban areas, respectively. An ST-segment elevation myocardial infarction (STEMI) shows a rapid onset. Among cardiovascular diseases, it is associated with the highest mortality [4]. According to a previous study, STEMI-related mortality during hospitalization of patients in China was 5.6%, and 30d mortality of males and females was 8.2% and 17.3%, respectively. The re-infarction rate was 11.2%, and the incidence of strokes was 2.5% [5]. Therefore, STEMIs place a great burden on patients and society [5].

Coronary vascular endothelial dysfunction leads to platelet activation, adhesion, aggregation, and thrombosis, causing severe occlusion of culprit vessels and the induction of severe myocardial ischemia or even necrosis in STEMI patients [6]. Today, antithrombotic therapy is the mainstay of STEMI treatment [7]. Considering that platelet activation plays a key role in the occurrence and development of STEMIs, antiplatelet drugs, including aspirin and clopidogrel, have become the cornerstone of STEMI treatment [6, 8].

Among antiplatelet drugs, clopidogrel is a widely used P2Y12 receptor antagonist in clinical treatment. However, clopidogrel has a number of imitations. It is a prodrug with a slow effect that requires cytochrome oxidase P450 to produce an active product through two steps of metabolism in the liver [9]. In addition, the cytochrome oxidase CYP2C19 allele is present in 25–35% of the population, and this genetic polymorphism can cause variability in the drug response, affecting its antiplatelet effect and resulting in drug resistance [10].

Ticagrelor, a P2Y12 receptor antagonist, is a new type of oral antiplatelet drug [11]. It is a non-prodrug, with rapid onset of action and reversibly binds to the P2Y12 receptor, which is conducive to hemostasis and emergency surgery [12]. The PLATO study showed that in acute coronary syndrome patients treated with ticagrelor for 1 y, the risk of composite endpoint events (i.e., cardiovascular death, myocardial infarctions, and strokes) was reduced, without increasing major bleeding events as compared with those in patients treated with clopidogrel [13, 14]. However, the PLATO study focused on clinical events after the application of ticagrelor, without considering the inhibition rate of platelet aggregation. It remains to be determined whether excessive platelet inhibition could lead to additional complications, such as bleeding.

In our previous study, we reported that ticagrelor decreased the occurrence rate of thrombosis, as well as ischemic outcome events, without causing any obvious increase in the risk of bleeding in acute STEMI patients receiving urgent percutaneous coronary intervention (PCI) [15]. In this study, we investigated the short-term efficacy (platelet inhibition) of ticagrelor and clopidogrel in acute ST-segment elevation myocardial infarction (STEMI) patients undergoing an emergency percutaneous coronary intervention (PCI). The findings can provide a scientific basis for the clinical safety and rational use of drugs.

## RESULTS

### Comparison of myocardial infarction-related indexes in the two groups

There were no statistically significant differences in the baseline characteristics of the ticagrelor and clopidogrel groups (Table 1). There were also no significant between-group differences in the infarction site, infarct-related vascular location, coronary artery opening time, maximum balloon expansion pressure, implant stent length and diameter, and TIMI risk score (*P* > 0.05; Table 2).

**Table 1:** Comparison of the baseline characteristics in the two groups.

	**Ticagrelor group (N = 49)**	**Clopidogrel group (N = 48)**	***P***
Age (year)	61.59 ± 11.22	62.79 ± 11.37	0.602
Gender (male/female)	30/19	29/19	0.935
BMI (kg/m^2^)	25.09 ± 2.53	24.09 ± 2.51	0.054
TC (mmol/L)	4.65 ± 0.96	4.76 ± 0.84	0.566
LDL-C (mmol/L)	3.18 ± 0.60	3.17 ± 0.49	0.909
TG (mmol/L)	1.41 ± 0.51	1.37 ± 0.50	0.690
AST (U/L)	39.22 ±7.55	39.71 ±6.36	0.734
ALT (U/L)	34.24 ± 5.98	35.85 ±5.78	0.181
CREA (umol/L)	89.23 ± 17.85	91.91 ± 19.16	0.477
Urea nitrogen (mmol/L)	7.16 ± 2.41	7.09 ± 2.16	0.880
Blood glucose under stress (mmol/L)	7.37 ± 2.83	8.40 ±2.22	0.051
cTnI (ng/mL)	3.39 ± 1.30	3.28 ± 1.36	0.689
BNP (pg/mL)	211.18 ± 27.25	216.08 ± 36.87	0.458
EF	0.49 ± 0.06	0.52 ± 0.08	0.073

BMI: body mass index. TC: total cholesterol. LDL: low-density lipoprotein. TG: triglyceride. AST: aspartate aminotransferase. ALT: alanine aminotransferase. CREA: creatinine. cTnI: troponin I. BNP: brain natriuretic peptide. EF: ejection fraction.

**Table 2:** Comparison of myocardial infarction-related indexes in the two groups.

	**Ticagrelor group (N = 49)**	**Clopidogrel group (N = 48)**	***P***
Infarction site [n (%)]			
Anterior myocardial infarction	25 (51.0)	23 (47.9)	0.760
Non-anterior myocardial infarction	24 (49.0)	25 (52.1)	0.760
Infarct-related vascular location [n (%)]			
Anterior descending branch	25 (51.0)	25 (52.1)	0.917
Circumflex artery	8 (16.3)	8 (16.7)	0.964
Right coronary artery	16 (32.7)	15 (31.3)	0.882
Coronary artery opening time (h)	4.23 ± 0.94	4.58 ± 1.12	0.101
Maximum balloon expansion pressure (atm)	10.69 ± 2.16	10.50 ± 2.06	0.653
Implant stent length (mm)	21.53 ± 5.87	21.50 ± 4.83	0.978
Implant stent diameter (mm)	2.93 ± 0.27	2.92 ± 0.32	0.845
High-risk TIMI risk score [n (%)]	21 (42.9)	19 (39.6)	0.743
Femoral artery path [n (%)]	9 (18.4)	10 (20.8)	0.760

TIMI: thrombolysis in myocardial infarction.

### Comparison of perfusion indicators of culprit vessels in the two groups

As shown in Table 3, the incidence of TIMI grade III blood flow in the ticagrelor group was significantly higher than that in the clopidogrel group (*P* < 0.05). There was no statistically significant difference in the incidence of TIMI grades 0–II blood flow in the two groups (*P* > 0.05). However, the corrected TIMI frame count (CTFC) in the anterior descending, circumflex, and right coronary arteries was statistically significantly lower in the ticagrelor group as compared with that in the clopidogrel group (*P* < 0.05).

**Table 3:** Comparison of thrombolysis in myocardial infarction blood flow, corrected TIMI frame count, and stent thrombosis after percutaneous coronary intervention in the two groups.

	**Ticagrelor group (N = 49)**	**Clopidogrel group (N = 48)**	***P***
TIMI blood flow [n (%)]			
TIMI grade 0	0 (0)	1 (2.1)	0.495
TIMI grade I	0 (0)	2 (4.2)	0.242
TIMI grade II	1 (2.0)	5(10.4)	0.111
TIMI grade III	48 (98.0)	40 (83.3)	0.016
CTFC, frames (n)			
Anterior descending branch	23.36 ± 2.68 (25)	25.24 ± 3.76 (25)	0.047
Circumflex	24.13 ± 4.94 (8)	30.50 ± 5.54 (8)	0.029
Right coronary artery	21.13 ± 4.18 (16)	25.47 ± 3.66 (15)	0.005
Acute stent thrombosis [n (%)]			
Definite	0 (0)	0 (0)	-
Probable	0 (0)	1 (2.1)	0.495
Subacute stent thrombosis [n (%)]			0.495
Definite	0 (0)	0 (0)	-
Probable	0 (0)	1 (2.1)	0.495
All stent thrombosis [n (%)]	0 (0)	2 (4.2)	0.242

TIMI: thrombolysis in myocardial infarction. CTFC: corrected TIMI frame count.

### Comparison of the incidence of stent thrombosis after the PCI in the two groups

During the follow-up, there was only one case of definite or probable acute stent thrombosis in the clopidogrel group and no cases in the ticagrelor group. There was also only one case of definite or probable subacute stent thrombosis in the clopidogrel group and no cases in the ticagrelor group. There was no significant between-group difference in the incidence of acute stent thrombosis, subacute stent thrombosis, or total stent thrombosis (*P* > 0.05; Table 3).

### Comparison of the antiplatelet effect of the drug treatment in the two groups

There were no statistically significant differences in the PLT count, MPV, and PDW in the ticagrelor and clopidogrel groups at three different measurements times: immediately after admission and 2 h and 7 d postdrug treatment (*P* > 0.05; Table 4).

**Table 4:** Comparison of the platelet count, mean platelet volume, and platelet distribution width in the two groups.

	**Ticagrelor group (N = 49)**	**Clopidogrel group (N = 48)**	***P***
PLT count (*10^9^/L)			
Immediately after admission	170.33±41.09	165.92±39.57	0.590
2 h after taking the medicine	167.45±45.33	160.69±41.48	0.447
7 days after taking the medicine	212.27±53.48	202.08±49.26	0.330
MPV (fL)			
Immediately after admission	11.42±1.24	11.62±1.08	0.410
2 h after taking the medicine	11.91±1.43	12.17±1.11	0.327
7 days after taking the medicine	10.61±1.16	10.63±1.02	0.940
PDW (%)			
Immediately after admission	16.26±3.25	16.04±3.07	0.743
2 h after taking the medicine	16.24±3.11	16.75±3.14	0.426
7 days after taking the medicine	14.20±2.64	14.20±3.10	1

PLT: platelet. MPV: mean platelet volume. PDW: platelet distribution width.

Thromboelastography (TEG) was used to evaluate the antiplatelet effect of the drugs. There were no significant between-group differences in the AA% and MA_AA_ at the three detection moments (*P* > 0.05; Table 5). There was no significant between-group difference in the ADP% and MA_ADP_ immediately after admission (*P* > 0.05). However, 2 h and 7 d after the drug treatment, the ADP% in the ticagrelor group increased significantly and the MA_ADP_ decreased significantly as compared with these parameters in the clopidogrel group (*P* < 0.05).

**Table 5:** Thromboelastography evaluation of the antiplatelet effect.

	**Ticagrelor group (N = 49)**	**Clopidogrel group (N = 48)**	***P***
AA%			
Immediately after admission	22.23 ± 7.35	21.35 ± 6.29	0.528
2 h after taking the medicine	69.15 ± 18.68	65.84 ± 17.84	0.374
7 days after taking the medicine	64.49 ± 16.92	59.39 ± 15.96	0.130
ADP%			
Immediately after admission	30.44 ± 11.38	31.21 ± 9.71	0.721
2 h after taking the medicine	79.18 ± 14.41	34.03 ± 11.04	< 0.001
7 days after taking the medicine	70.62 ± 11.50	53.77 ± 10.37	< 0.001
MA_AA_			
Immediately after admission	81.00 ± 11.99	82.38 ± 10.40	0.546
2 h after taking the medicine	32.35 ± 10.46	32.91 ± 9.48	0.783
7 days after taking the medicine	37.50 ± 10.45	34.46 ± 8.82	0.126
MA_ADP_			
Immediately after admission	74.86 ± 12.85	75.27 ± 13.06	0.877
2 h after taking the medicine	21.57 ± 9.67	64.13 ± 13.68	< 0.001
7 days after taking the medicine	34.89 ± 7.11	42.11 ± 13.19	0.001

AA%: arachidonic acid (AA)-induced platelet inhibition rate. ADP%: adenosine diphosphate (ADP)-induced platelet inhibition rate. MA_AA_: platelet aggregation rate of the AA pathway. MA_ADP_: platelet aggregation rate of the ADP pathway.

### Comparison of complications and side effects in the two groups

As shown in Table 6, there were no cases of bradycardia in either group and no statistically significant difference in the incidence of hemorrhages in the two groups (*P* > 0.05). However, the incidences of dyspnea and kidney failure in the clopidogrel group were clearly lower than those in the ticagrelor group (*P* < 0.05).

**Table 6:** Comparison of complications and side effects in the two groups.

	**Ticagrelor group (N = 49)**	**Clopidogrel group (N = 48)**	***P***
Dyspnea [n (%)]	6(12.2)	0 (0)	0.027
Bradycardia [n (%)]	0 (0)	0 (0)	-
Kidney failure [n (%)]	8 (16.3)	0 (0)	0.006
Hemorrhage [n (%)]	2 (4.1)	1 (2.1)	1.000

### Comparison of ischemic endpoint events in the two groups

Follow-up of ischemic endpoints during hospitalization and within 30 d postdischarge in both groups revealed no statistically significant between-group differences in cardiac death, nonfatal myocardial infarction, and emergency coronary revascularization (*P* > 0.05, Table 7).

**Table 7:** Comparison of ischemic endpoint events in the two groups.

	**Ticagrelor group (N = 49)**	**Clopidogrel group (N = 48)**	**P**
Cardiac death	0 (0)	1 (2.1)	0.495
Nonfatal myocardial infarction	1 (2.0)	2 (4.2)	0.617
Emergency coronary revascularization	1 (2.0)	1 (2.1)	1.000

## DISCUSSION

The primary treatment for patients with an acute STEMI is the early opening of the infarct-related blood vessels, which restores reperfusion of blood flow in the infarcted area, reduces the infarct size, prevents ventricular remodeling, and eventually improves the patient’s short-term and long-term prognosis [16]. There are three main methods for opening the culprit vessels: intravenous thrombolysis, coronary artery bypass grafting, and PCI. Among these, an emergency PCI serves as first-line revascularization treatment due to characteristics of a low level of trauma, quick recovery, and patient acceptance, [17].

The TIMI blood flow grading system is an effective method for evaluating vascular perfusion in myocardial infarction patients. According to this system, TIMI grade III blood flow denotes normal perfusion, whereas TIMI grades 0–II signify no/slow reflow. In PCI, due to balloon expansion and stent placement, strong pressure is exerted on the coronary vessel wall, which damages the intima of the blood vessel, activates platelets, produces thrombus, and occludes the distal blood vessels to block TIMI blood flow, eventually leading to a reduction of myocardial perfusion. Therefore, antithrombotic therapy is key [18]. Our study showed that the incidence of TIMI grade III blood flow in the ticagrelor group was significantly higher than that in the clopidogrel group, which was inconsistent with the findings of the PLATO study where the incidence of TIMI grade III blood flow was similar in the ticagrelor- and clopidogrel-treated groups [19]. The discord in the results might be due to the proportion of acute STEMI patients in the two studies: Fewer than 50% of patients had an acute STEMI in the invasive treatment subgroup in the PLATO study, whereas all the patients in the present study had an acute STEMI and underwent a PCI. The absence of a statistically significant difference in the incidence of TIMI grades 0–II blood flow in the two groups in the present study may be due to the small sample size. Overall, this study suggested that ticagrelor significantly improved TIMI blood flow than clopidogrel in STEMI patients undergoing an emergency PCI.

It should be noted that TIMI blood flow grading has limitations, including poor reproducibility and dependency on the clinical experience of the operator. In contrast to the TIMI grading system, the CTFC provides a simple, easy, objective, and accurate method for evaluation of the blood flow of culprit vessels. Previous research demonstrated that the CTFC was negatively correlated with the cardiac ejection fraction value in acute STEMI patients during hospitalization and that it was positively correlated with adverse cardiac events [20]. In the present study, there was a statistically significant between-group difference in the CTFC of the anterior descending, circumflex, and right coronary arteries, and the CTFC in the ticagrelor group was significantly better than that in the clopidogrel group, suggesting that the preoperative loading dose of ticagrelor has a significant advantage over clopidogrel in improving myocardial infarction. This advantage may be related to the stronger and faster antiplatelet pharmacological mechanism of ticagrelor [21].

The results of the present study also suggested that the total stent thrombosis rate in the ticagrelor group (0) was lower than that in the clopidogrel group (4.2%) within 30 d after discharge, which was consistent with the findings of the PLATO study [14]. Although there is no statistically significant difference in the total stent thrombosis rate in our study, most likely due to the small sample size, these results still confirmed the clinical significance of ticagrelor in acute STEMI patients undergoing a PCI.

Antiplatelet drugs are critical for the treatment of acute STEMIs. Clinical evaluation of platelet aggregation function can indicate the efficacy of antiplatelet drugs and guide specialists in individualized treatment of patients. Currently, light transmission platelet aggregation (LTA) is the most widely used method for platelet function detection and considered the gold standard [22]. Light transmission platelet aggregation (LTA) measures the response of platelets to ADP to evaluate the inhibition rate of platelets by antiplatelet drugs [23]. However, controversy surrounds the use of the LTA measurement because of shortcomings, such as the need for special laboratory equipment, complicated specimen preparation, poor reproducibility, a long detection process, and low specificity for P2Y12 channels.

Thromboelastography (TEG) is a new type of detection method that can evaluate the whole process of platelet aggregation in the blood [24, 25]. It has the following advantages over LTA. First, it can simulate the whole coagulation process *in vitro*, reflecting interactions between the various coagulation components more directly and comprehensively; second, whole blood specimens can be rapidly measured at the bedside without special treatment, with high contrast and good reproducibility; finally, the results are automatically analyzed and processed using a computer analyzer and can be quickly printed for clinical reference. Many studies have demonstrated the reliability of TEG for evaluating platelet aggregation [26, 27]. The CREST study, which included 150 patients with clopidogrel resistance who underwent an elective PCI, suggested that TEG could clinically guide the development of individualized antiplatelet regimens and drug efficacy monitoring and no stent thrombosis occurred during follow-up by rationally adjusting antiplatelet agents [27]. In this study, we observed no significant differences in routine platelet analysis (PLT count, MPV, and PDW) of the patients in the ticagrelor and clopidogrel groups, suggesting that the two drugs had no significant effect on the number and volume of platelets. When we evaluated the effect of antiplatelet drugs in the two groups using TEG, the ADP% in the ticagrelor group was significantly higher than that in the clopidogrel group, which was consistent with the findings of a previous study [28]. In the present study, the MA_ADP_ in the ticagrelor group was significantly decreased as compared with that in the clopidogrel group. These results confirm that ticagrelor is more effective than clopidogrel, with a rapid, long-lasting, strong, and stable antiplatelet aggregation effect. The results also suggested that the ADP% or MA_ADP_ in TEG can be used as a reliable indicator of efficacy in evaluations of antiplatelet drugs. In addition, this study also proves that TEG can quickly and accurately detect the platelet aggregation rate to evaluate platelet inhibition by antiplatelet drugs, which is beneficial in the clinical selection of types and doses of antiplatelet drugs.

In the present study, we also compared complications and ischemic endpoint events in the ticagrelor and clopidogrel groups. We found no statistically significant differences in the incidences of hemorrhages and bradycardia, cardiac deaths, nonfatal myocardial infarctions, and emergency coronary revascularization in the two groups, supporting the clinical application of ticagrelor.

The present study has some limitations. First, the collected samples were all from cardiology inpatients from a single center. Thus, the results represent only those of the hospitalized patients in this center. In addition, the sample size was relatively small, which may have led to some bias in the statistical results, especially in terms of complications, side effects, and ischemic endpoint events. Furthermore, the follow-up time was only 30 d. Finally, dynamic changes in the relevant indicators in the two groups were determined at selected time points. Dynamic changes at multiple time points may be more valuable for clinical guidance. These shortcomings will be addressed in a later study.

In conclusion, this study focused on the antiplatelet effect of ticagrelor and clopidogrel in acute STEMI patients who underwent an emergency PCI. Compared with clopidogrel, ticagrelor significantly improved and decreased the incidence of TIMI grade III blood flow and CTFC in patients, respectively, indicating that ticagrelor can reduce the occurrence of no- and slow-reflow as compared with that of clopidogrel. Furthermore, in terms of its antiplatelet effect, ticagrelor was faster, stronger, and longer-lasting than clopidogrel.

## MATERIALS AND METHODS

### Subjects

This study was approved by the ethics committee of Yantai Affiliated Hospital of Binzhou Medical University, China and Xuzhou Central Hospital, China. All patients provided signed informed consent forms.

In total, 97 STEMI patients (males, *n* = 52; females, *n* = 45) who were admitted to our hospital between December 2013 and May 2015 were sequentially enrolled. These patients underwent emergency coronary angiography (CAG) and PCI. The age range of the patients was 38–75 y (62.19 ± 11.25 y). The patients were assigned random numbers according to the hospital admission time, and these random numbers were then sorted using a computer-generated random number sequence in a table. Based on the treatment, the patients were divided into two groups: a ticagrelor group (*n* = 49) and a clopidogrel group (*n* = 48). The patients were followed up by telephone within 30 d after discharge.

According to a two-sample, two-sided equality test [29], 48 patients in each group will have power of 97.51 in estimating a 20% difference in the incidence of thrombolysis in myocardial infarction (TIMI) grade III blood flow. The inclusion of 48 patients in each group made it possible to detect a significant difference at the levels of α = 0.05 and β = 0.0249.

### Inclusion criteria

The inclusion criteria were as follows: i) patients aged 38–75 y, with indications for emergency PCI surgery; ii) patients meeting diagnostic criteria of acute myocardial infarction stipulated by the Chinese Society of Cardiology (2010); iii) patients with STEMI onset of no more than 12 h or with chest tightness and persistent ST-segment elevation after 12-h onset; iv) acute myocardial infarction patients who had not undergone revascularization for nonculprit vascular lesions, including lesions in other parts of culprit vessels and non-culprit vessels within 30 d; and v) patients who agreed to participate in this clinical trial at the time of admission and signed written informed consent before surgery.

### Exclusion criteria

The exclusion criteria were as follows: i) patients with severe adverse reactions to ticagrelor or resistance to clopidogrel or allergies to contrast agents; ii) patients who were not suitable for a PCI according to their clinical experience, previous examinations, and CAG diagnosis; iii) patients with left main disease, cardiogenic shock, and an acute STEMI within 24 h of thrombolysis; iv) patients with a history of PCIs or coronary artery bypass grafting; v) patients with a high risk of bleeding or active bleeding or surgery or trauma within 3 mo prior to enrollment; vi) patients who had taken proton pump inhibitors or H_2_ antagonists within 1 wk prior to enrollment; vii) patients with abnormal findings on routine blood work (hemoglobin of < 100 g/L and platelet count of < 100 × 10^9^/L); viii) patients with a previous myocardial infarction or other heart disease and/or severe heart failure (New York Heart Association class III–IV); ix) patients with endocrine diseases, such as diabetes, digestive tract ulcers, acute and chronic infections, malignant tumors, blood diseases, and immune system diseases (e.g., rheumatic disease of connective tissues); x) patients with cerebrovascular diseases and peripheral vascular diseases; xi) patients with severely abnormal liver and kidney function (i.e., an increase in alanine aminotransferase and aspartate aminotransferase of more than three times the normal levels due to a non-ST-elevation myocardial infarction; a serum creatinine level of more than180 umol/L); xii) patients with a history of heparin-induced thrombocytopenia; xiii) patients with severely uncontrolled hypertension (systolic blood pressure ≥ 180 mmHg and/or diastolic blood pressure ≥ 110 mmHg); and xiv) patients who had any conditions deemed inappropriate by the investigators for inclusion in the study (e.g., late presenters where the efficacy of antiplatelet therapy was substantially diminished).

### Drug treatment

### Standardized use of antiplatelet drugs before the PCI

The patients in the ticagrelor group received 300 mg of aspirin (Bayer, Germany) and 180 mg of ticagrelor (AstraZeneca, UK) once daily, and the patients in the clopidogrel group received 300 mg of aspirin and 600 mg of clopidogrel (Sanofi, France) once daily.

### Standardized use of antiplatelet drugs after the PCI

The patients in the ticagrelor group took 100 mg of aspirin once daily, in addition to 90 mg of ticagrelor twice daily, for 12 mo after surgery.

The patients in the clopidogrel group took 100 mg of aspirin once daily, in addition to 75 mg of clopidogrel once daily, for 12 mo after surgery.

### Related drug treatment

Patients with specific indications were treated with low-molecular-weight heparin, IIb/IIIa glycoprotein inhibitors, β-receptor inhibitors, angiotensin-converting-enzyme inhibitors/angiotensin receptor blockers, statins, calcium channel blockers, nitrates, and proton pump inhibitors/ H_2_ antagonists.

### Coronary interventional therapy (CAG plus PCI)

The patient’s radial artery was used with the needle inserted at the most obvious pulsation according to the Seldinger method. With the guidance of the puncture needle, the guide wire was set and then fed into the arterial dilatation sheath, followed by an injection of 3,000 U heparin sodium into the outer sheath tube for anticoagulation. Catheters were then delivered along the sheath to the left and right coronary openings under the guidance of J-shaped guide wire. In left coronary artery, contrast agent (iohexol, 350 mg/ml) was administered in five projections (spider, foot, liver, head, and right shoulder) and in the right coronary artery, contrast agent was administered at least in the left anterior oblique and head projections. If necessary, other positions can be added to achieve the full display of the coronary artery segments. The experienced clinical cardiologists performed all the operations. Two experienced cardiac intervention specialists evaluated the following parameters: the degree of coronary artery stenosis, using the internationally accepted visual method, which is calculated as the percentage of reduction in the diameter of stenosis as compared with the diameter of normal coronary vessels at the proximal and distal ends.

The degree of coronary artery stenosis is calculated using the internationally accepted visual method, which is calculated as the percentage of the diameter of the stenosis that is smaller than the decrease in the diameter of the normal coronary artery at the concentric and distal end of the adjacent stenosis. All results were based on consensus. The observation indexes are shown in Supplementary Table 1.

### Blood specimen collection

Elbow venous blood (5 ml) was collected immediately after admission prior to any drug treatment and again 2 h after the drug treatment. Another 5 ml of fasting elbow venous blood was collected 7 d after the drug treatment. Following the blood collection, a tourniquet was not applied, and tapping the blood vessels at the puncture site was avoided. The collected blood specimen was not subjected to any violent oscillations to reduce potential damage to platelets and avoid hemolysis of the specimen.

### Determination of relevant detection indicators

### Platelet determination

Using an XE-2100D blood analyzer (SYSMEX, Japan) and associated reagents, the platelet (PLT) count, mean platelet volume (MPV) [30], and platelet distribution width (PDW) were determined immediately after admission and 2 h and 7 d after the drug treatment.

### Thromboelastography determination

Thromboelastography (TEG) was performed using a thromboelastography analyzer (TEG-5000; Haemoscope Corporation, USA) and platelet aggregation detection kit (coagulation method) according to the instructions of the manufacturer. Briefly, immediately after admission and 2 h and 7 d postdrug treatment, 5 ml of elbow venous blood was placed in a citric acid tube. Then, 1 ml of blood was added to the tube containing kaolin and mixed gently. Subsequently, 360 μl of the kaolin-treated blood sample was added to a preheated test cup containing 20 μl of 0.2 M CaCl_2_. The total clot strength (MAthrombin) of thrombin-induced platelet activation was measured using the TEG coagulation analyzer. Briefly, the blood sample was placed in a static cylindrical stainless steel cup in which the coagulation analyzer automatically adjusted the temperature to remain at 37° C and then rotated at an angle of 4°45', with each rotation lasting 10 sec. The blood sample was monitored by immersing the small stainless steel cylinder in the blood sample and the sensor that was connected to the cylinder. When the blood sample was in a liquid state, the rotation of the cup did not affect the inner cylinder, and the signal reflected on the tracing paper by the sensor was a straight line. When the blood coagulated, the movement amplitude of the small stainless steel cylinder was affected by the strength of the fibrin-platelet complex. The rotation of the cup drove the cylinder to move at the same time; as the internal resistance continued to increase, the movement of the built-in cylinder driven by the cup gradually weakened. This process was converted and recorded by the sensor. The data were then automatically analyzed and processed using a computer, generating the TEG.

Then, prepared activator F (10 μl) was added to two blank test cups, followed by the addition of 10 μl of arachidonic acid (AA) and adenosine diphosphate (ADP) reagents, respectively. In total, 360 μl of heparinized whole blood was placed in two test cups and mixed to form a whole blood cross-linked clot activated by platelet activator. The AA-induced platelet inhibition rate (AA%), platelet aggregation rate of the AA pathway (MA_AA_), ADP-induced platelet inhibition rate (ADP%), and platelet aggregation rate of the ADP pathway (MA_ADP_) were then obtained using the TEG analyzer and associated computer software according to the method described above to evaluate the effects of the drug treatments (i.e., aspirin, clopidogrel, and ticagrelor) on platelet function.

### Statistical analysis

SPSS version 22.0 statistical software was used for data analysis. The measurement data were expressed as mean ± standard deviation (SD). For data that conformed to a normal distribution, differences between the two groups were analyzed using an independent samples *t*-test. For data with a non-normal distribution, the Mann–Whitney U test was used for the analysis of between-group differences. The enumeration data are shown as cases/percentage (*n*/%) and analyzed using a chi-square (χ^2^) test or Fisher’s exact probability analysis. A value of *P* < 0.05 was considered statistically significant.

## Supplementary Material

Supplementary Table 1
