# Supplementary material for: Short-term efficacy of ticagrelor in acute ST-segment elevation myocardial infarction patients undergoing an emergency percutaneous coronary intervention
Source: Aging (Albany NY). 2019 Oct 30;11(20):8925–36. doi: 10.18632/aging.102353 (PMC6834424; doi:10.18632/aging.102353)
Supplement: Supplementary Table 1 [file aging-11-102353-s001.pdf]

SUPPLEMENTARY TABLE

Supplementary Table 1. The observation indexes used in this study.

Observation indexes	Description
Infarction site	Anterior myocardial infarction and non-anterior myocardial infarction.
Infarct-related vascular location	Anterior descending branch, circumflex artery, and right coronary artery.
Coronary artery opening time	The time from onset to balloon expansion or stent placement.
Maximum balloon expansion pressure	The pressure at which the balloon was maximally expanded
Implant stent length and diameter	
TIMI risk score	The patients with the TIMI risk score ≥ 5 were divided into the high-risk group [1].
Femoral or radial artery path	
Myocardial microcirculation perfusion indexes	Coronary TIMI blood flow [2–4]: TIMI grade 0: no perfusion; TIMI grade I: micro-perfusion; TIMI grade II: partial perfusion; TIMI grade III: complete perfusion.
	TIMI Frame Count (TFC) [5]: it is defined as the number of frames required for the coronary artery from the begin of coloring to normalized distal marker development during coronary angiography. Due to the long left anterior descending coronary artery, a correction factor (1.7) is required to compensate for the difference between the circumflex and right coronary artery. After the correction of the length of the blood vessel, the result was called the corrected TIMI Frame Count (CTFC). The international common frame number (30 frames/s) was used.
Stent thrombosis	Definite stent thrombosis is confirmed by contrast and pathological examination; probable stent thrombosis refers to any unexplained death or myocardial infarction of the patient within 30 days; acute stent thrombosis occurs within 24 h; subacute stent thrombosis occurs from 24 h to 30 days.
Complications and ischemic endpoint events	Complications: dyspnea, bradycardia, kidney failure, and hemorrhage.
	Ischemic endpoint events: cardiac death, nonfatal myocardial infarction, and emergency coronary revascularization.

1. Scirica BM, Cannon CP, Antman EM, Murphy SA, Morrow DA, Sabatine MS, McCabe CH, Gibson CM, Braunwald E. Validation of the thrombolysis in myocardial infarction (TIMI) risk score for unstable angina pectoris and non-ST-elevation myocardial infarction in the TIMI III registry. Am J Cardiol. 2002; 90:303–5. https://doi.org/10.1016/S0002-9149(02)02468-2

2. Kammler J, Kypta A, Hofmann R, Kerschner K, Grund M, Sihorsch K, Steinwender C, Lambert T, Helml W, Leisch F. TIMI 3 flow after primary angioplasty is an important predictor for outcome in patients with acute myocardial infarction. Clin Res Cardiol. 2009; 98:165–70. https://doi.org/10.1007/s00392-008-0735-9 PMID:18975020

3. Ndrepepa G, Mehilli J, Tiroch K, Fusaro M, Kufner S, Ellert J, Goedel J, Schomig A, Kastrati A. Myocardial perfusion grade, myocardial salvage indices and long-term mortality in patients with acute myocardial infarction and full restoration of epicardial blood flow after primary percutaneous coronary intervention. Rev Esp Cardiol. 2010; 63:770–8. https://doi.org/10.1016/S0300-8932(10)70179-2

4. Stone GW, Cox D, Garcia E, Brodie BR, Morice MC, Griffin J, Mattos L, Lansky AJ, O'Neill WW, Grines CL. Normal flow (TIMI-3) before mechanical reperfusion therapy is an independent determinant of survival in acute myocardial infarction: analysis from the primary angioplasty in myocardial infarction trials. Circulation. 2001; 104:636–41. https://doi.org/10.1161/hc3101.093701 PMID:11489767

5. Gibson CM, Cannon CP, Daley WL, Dodge JT, Jr., Alexander B, Jr., Marble SJ, McCabe CH, Raymond L, Fortin T, Poole WK, Braunwald E. TIMI frame count: a quantitative method of assessing coronary artery flow. Circulation. 1996; 93:879–88. https://doi.org/10.1161/01.CIR.93.5.879 PMID:8598078
